# Is magnetic resonance/ultrasound fusion prostate biopsy better than systematic prostate biopsy? an updated meta- and trial sequential analysis

**DOI:** 10.18632/oncotarget.6201

**Published:** 2015-10-20

**Authors:** Jian Wu, Alin Ji, Bo Xie, Xiao Wang, Yi Zhu, Junyuan Wang, Yasai Yu, Xiangyi Zheng, Ben Liu, Liping Xie

**Affiliations:** ^1^ Department of Urology, The First Affiliated Hospital, School of Medicine, Zhejiang University, Hangzhou, Zhejiang Province, People's Republic of China; ^2^ Department of Urology, Tongde Hospital of Zhejiang Province, Hangzhou, Zhejiang Province, People's Republic of China

**Keywords:** prostate cancer, magnetic resonance imaging, prostate biopsy, target biopsy, meta-analysis

## Abstract

We systematically reviewed the literature to determine whether Magnetic Resonance/Ultrasound (MR/US) fusion prostate biopsy is better than systematic biopsy for making a definitive diagnosis of prostate cancer. The two strategies were also compared for their ability to detect lesions with different degrees of suspicion on MRI and clinically significant prostate cancer, and the number of cores needed for diagnosis. The Cochrane Library, Embase, Web of Knowledge, and Medline were searched from inception until May 1, 2015. Meta-analysis was conducted via RevMan 5.2 software. Data was expressed as risk ratio (RR) and 95% confidence interval. Trial sequential analysis was used to assess risk of random errors. Fourteen trials were included, encompassing a total of 3105 participants. We found that MR/US fusion biopsy detected more prostate cancers than systematic biopsy (46.9% *vs*. 44.2%, *p*=0.03). In men with moderate/high MRI suspicion, MR/US fusion biopsy did better than systematic biopsy (RR = 1.46; *p* < 0.05) for making a diagnosis. Moreover, MR/US fusion biopsy detected more clinically significant cancers than systematic biopsy (RR = 1.19; *p* < 0.05). We recommend that MR/US fusion prostate biopsy be used to better detect prostate cancer, particularly in patients with moderate/high suspicion lesions on MRI.

## INTRODUCTION

Prostate cancer accounts for 10% of all cancer-related deaths in the United States [1]. Prostate cancer is usually suspected when an abnormality is felt during a digital rectal examination (DRE) and levels of prostate specific antigen (PSA) are elevated. However, a definitive diagnosis depends on the histopathological verification of adenocarcinoma via prostate biopsy cores or operative specimens. The systematic biopsy protocol under transrectal ultrasound (TRUS) has been the standard procedure for detecting prostate cancer for decades. Nevertheless, it has a high false negative rate, with estimates as high as 47% [2]. Furthermore, systematic prostate biopsy misses 50% to 80% of clinically significant prostate cancers [3]. A new imaging technology is necessary to better diagnose prostate cancer.

Fusion-weighted magnetic resonance imaging (mp-MRI) achieves a higher diagnostic rate and lower false-negative rate when used in prostate cancer patients. Mp-MRI used in conjunction with Magnetic Resonance/Ultrasound (MR/US) fusion guided biopsy is also helpful in establishing a correct diagnosis in men suspected of having prostate cancer, and is both efficient and cost-effective. However, because the MR/US fusion biopsy is a new technology, it is still controversial as to whether it is a stable and accurate method for detection of prostate cancer [4-6], and whether it outperforms systematic biopsy [7,8]. Although, it was previously suggested that the two methods did not differ in overall prostate cancer detection, MR/US fusion biopsy had a higher rate of detection of clinically significant cancers [9,10].

The evidence base from trials has recently increased [11-13], with more support for the idea that MR/US fusion biopsy detects prostate cancer better than systematic biopsy. We therefore performed a systematic review to reevaluate the value of MR/US fusion biopsy. Moreover, since random error may skew meta-analysis results, we used trial sequential analysis (TSA) to reduce the risk of random errors in our study [14].

## RESULTS

### Description of meta-analysis

**Figure 1:**
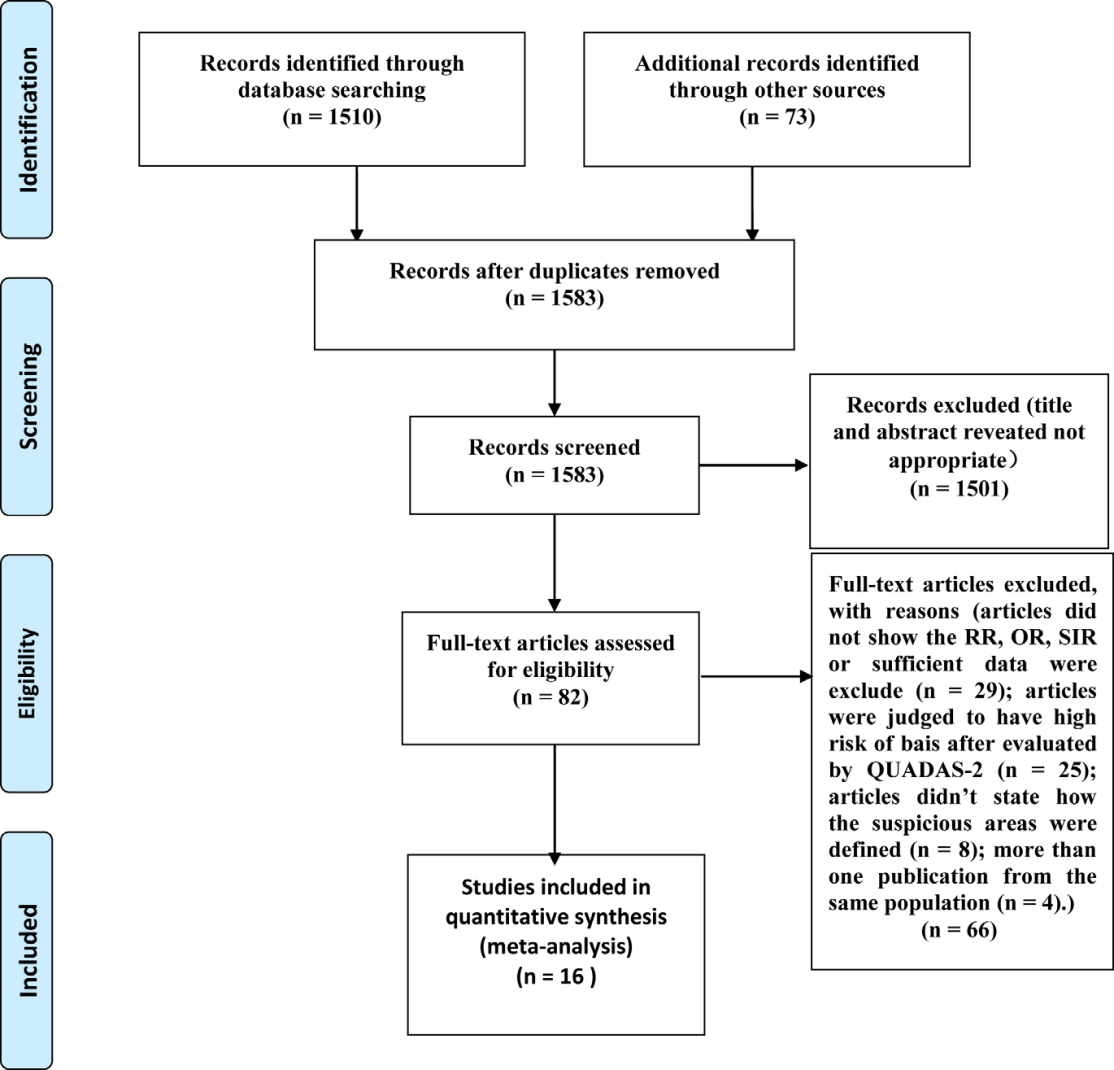
PRISMA (Preferred Reporting Items for Systematic reviews and Meta-Analyses) flow diagram showing an overview of the study selection process

The systematic search identified 1583 relevant references. After screening titles and abstracts, we excluded 1501 articles such as imaging studies, meeting abstracts, reviews, letters, and other articles irrelevant to our study. The 82 remaining articles were retrieved in full text for formal review. After assessing full text, 66 articles were excluded. After review, 16 paired cohort studies were included to compare the detection rate of prostate cancer between MR/US fusion biopsy and systematic biopsy. The systematic search has been done following the PRISMA statement, which is shown in appendix file 1. Exclusion criteria are shown in Figure 1, and details of the 16 included 16 studies is shown in Table 1.

**Table 1:** Characteristics of the fourteen included studies

Study	Country	Study design	No. of participants	Age. yr, mean (or median)	PSA.ng/ml, mean(or median)	Biopsy protocol		Definition for clinicallySignificant Pca	Score used in mp-MRI
						MR/US Fusion biopsy	System biopsies		
Ardeshir R. Rastinehad, 2014	USA	Cohort	105	65.8 (42-87)	9.5(0.6-62)	3.9 core	15.8 core	The Epstein criteria	Low, moderate and high
Gaell e Fiard, 2013	France	Cohort	30	64 (61-67)	6.3 (5.2-8.8)	2 core each lesion	12 core	Gleason≥3 + 4 or total cancer length≥10 mm	PI-RADS
Baco, E 2015	USA	RCT	175	65 (59-65)	7.3 (5.5–9.9)	2 core each lesion	12 core	Gleason≥3 + 4 or total cancer length ≥5 mm	PI-RADS
Geoffrey A. Sonn, 2013	USA	Cohort	105	65 (59-70)	7.5 (5.0-11.2)	2 core each lesion	12 core	Gleason≥3 + 4 or total cancer length ≥4 mm	5-point scale
de Gorski, A 2015	France	Cohort	232	64 ± 6.4	6.5 ± 1.8	2 or 3 core each lesion	12 core	Gleason≥3 + 4 or total cancer length ≥4 mm	Likert
Philippe Puech, 2013	France	Cohort	95	65 (49-76)	10.05±8.8	NA	12 core	Gleason≥3 + 4 or total cancer length≥3 mm	Likert
Pierre Mozer, 2014	France	Cohort	152	63±5.7	6±1.7	2 core each lesion	12 core	Gleason≥3 + 4 or total cancer length e≥4 mm	Likert
Srinivas Vourganti, 2012	USA	Cohort	195	62 (37-80)	9.13 (0.3-103)	5 core (2-14)	12 core	Gleason≥3 + 4	Low, moderate and high
Timur H. Kuru, 2013	Germany	Cohort	347	65.3 (42-82)	9.85 (0.5-104)	2 core each lesion	12 core	NCCN criteria	Not suspicious,questionable, andhighly suspicious
James S. Wysock, 2013	USA	Cohort	125	65 (56–71)	5.1 (3.5–7.3)	2 core each lesion	12 core	The Epstein criteria	5-point scale
Tomoaki Miyagawa, 2010	Japan	Cohort	85	69 (56–84)	9.9 (4–34.2)	1.9 core each lesion	12 core	NR	NR
Delongchamps, 2013	France	Cohort	133	64.5±7.9	9±3.9	3 core each lesion	12 core	Gleason≥3 + 4 or total cancer length ≥5 mm	Benign, intermediate,malignant
M. Minhaj Siddiqui, 2015	USA	Cohort	1003	62.1±7.5	6.7 (4.4-10.7)	5.3±2.6 core	12.3±0.7 core	NR	Low, moderate and high
Angelika Borkowetz, 2015	German	Cohort	263	66 (47-83)	8.3 (0.39-86.57)	8.9±2.7 core	12.3±1.5 core	The Epstein criteria	PI-RADS
Daniel Junker, 2015	Austria	Cohort	50	63.7±7.9	7.6±7.9	4.5±0.76 core	10 core	NR	PI-RADS
Osamu Ukimura, 2015	USA	Cohort	127	66 (39–81)	5.8 (1.4–28.8)	2.78 core	11.0 core	Gleason≥3 + 4 or total cancer length ≥5 mm	highly suspicious, likely suspicious,

### Assessment of quality and risk of bias

**Figure 2:**
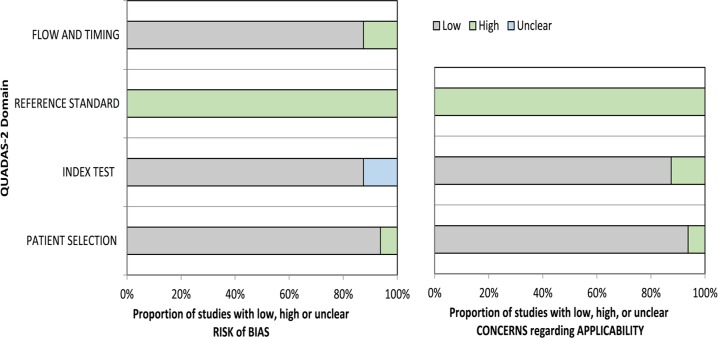
Summary of risk of bias assessment of all papers included using the quality assessment of Diagnostic Accuracy Studies-2 tool

While there was variation in the methodological quality of included studies (Table 2), none of them was judged to be at overall risk of bias because TRUS-guided prostate biopsy was chosen as a reference standard. Most of the other factors were judged as “low risk of bias” (Table 2; Figure 2).

**Table 2:** Risk of bias assessment of each study using the Quality Assessment for Diagnostic Studies-2 tool

Study	RISK OF BIAS				APPLICABILITY CONCERNS		
	PATIENT SELECTION	INDEX TEST	REFERENCE STANDARD	FLOW AND TIMING	PATIENT SELECTION	INDEX TEST	REFERENCE STANDARD
Ardeshir R. R, 2014	−	−	+	−	−	−	+
Gaell e Fiard, 2013	−	−	+	−	−	−	+
Baco, E 2015	−	−	+	−	−	−	+
Geoffrey A. Sonn, 2013	−	−	+	−	−	−	+
de Gorski, A 2015	−	−	+	−	−	−	+
Philippe Puech, 2013	−	+	+	−	−	+	+
Pierre Mozer, 2014	−	−	+	−	−	−	+
Srinivas Vourganti, 2012	−	−	+	−	−	−	+
Timur H. Kuru, 2013	?	+	+	?	+	−	+
James S. Wysock, 2013	−	−	+	−	−	−	+
Tomoaki Miyagawa, 2010	−	−	+	−	−	−	+
Delongchamps, 2013	−	−	+	?	−	−	+
M. Minhaj Siddiqui, 2015	−	−	+	−	−	−	+
Angelika Borkowetz, 2015	−	−	+	−	−	+	+
Daniel Junker, 2015	−	−	+	−	−	−	+
Osamu Ukimura, 2015	−	−	+	−	−	−	+

− Low Risk + High Risk ? Unclear Risk

### Overall analysis of the two different biopsy protocols

**Figure 3:**
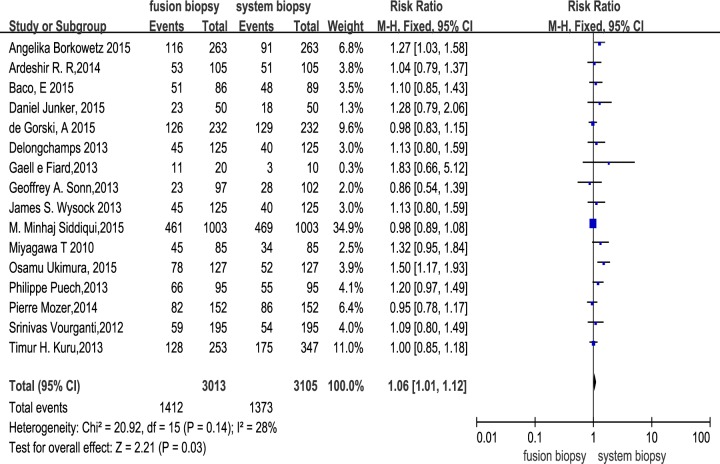
Effect of overall detection rate of prostate cancer with fusion biopsy and systematic biopsy

**Figure 4:**
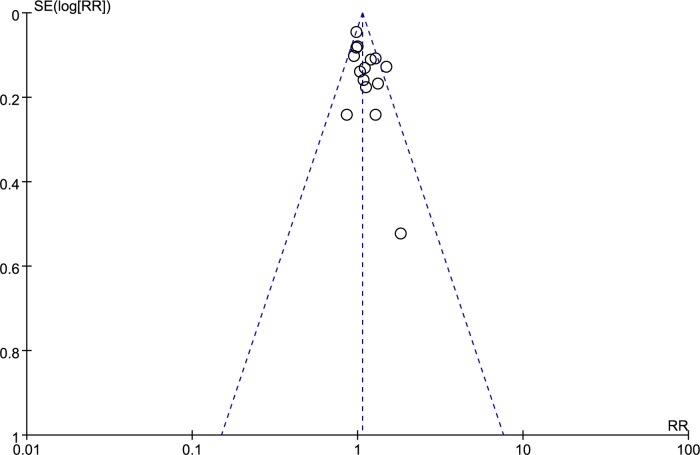
Funnel plots of overall detection rate of prostate cancer with MR/US fusion biopsy and systematic biopsy SE = standard error, RD = risk difference.

**Figure 5:**
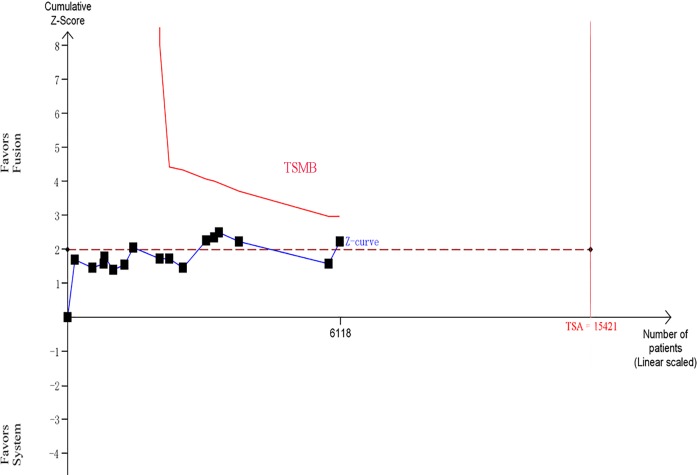
Trial sequential analysis of trials in overall analysis between two biopsy strategies The Z-curve only crosses the conventional boundary, and did not reach the trial sequential monitoring boundary (TSMB) and TSA information size.

Across the 16 trials, there were a total of 3105 participants who underwent MR/US fusion guided biopsy or TRUS-guided prostate biopsy. Prostate cancer was diagnosed in 1412 of the men with MR/US fusion biopsy compared with 1373 with the TRUS-guided systematic biopsy, resulting in a RR of 1.06 (95% CI, 1.01–1.12; *p* = 0.03; Table 3; Figure 3). There was moderate heterogeneity among these trials (I^2^ = 28%; x^2^ = 20.92; *p* = 0.14). The funnel plots revealed little publication bias in this overall analysis (Figure 4), and both the Egger's test and the Begg's test indicated there was no publication bias. In trial sequential analysis, the number of participants did not reach the informative size. The cumulative Z-curve approached cross-traditional significance boundaries but did not cross the trial sequential monitoring boundary (TSMB; Figure 5).

### Subgroup analysis of MRI suspicion

Subgroup analysis was based on four trials according to different degrees of MRI suspicion (low MRI suspicion, moderate suspicion, high suspicion; Table 3). Prostate cancer was diagnosed in 33 of the 253 men with low suspicion using MR/US fusion biopsy as compared with 219 of the 554 men with TRUS-guided systematic biopsy, resulting in a RR of 0.36 (95% CI, 0.26-0.49; *p* < 0.01; Table 3). Prostate cancer was diagnosed in 207 of the 365 men with moderate/high MRI suspicion using MR/US fusion biopsy compared with 219 of the 554 men with TRUS-guided systematic biopsy, resulting in a RR of 1.46 (95% CI, 1.28–1.67; *p* < 0.01; Table 3).

**Table 3:** Stratified analyses of different biopsy schemes

	n	Sample size*	Heterogeneity		MR/US fusion biopsy vs System biopsies	
			P_h_	I^2^, %	RR (95%CI)	*p*
Total	16	3013/3105	0.14	28	1.06 (1.01, 1.12)^a^	0.03^a^
					1.09 (1.01, 1.18)^b^	0.02^b^
Low MRI suspicion	4	253/554	0.05	63	0.36 (0.26, 0.49)	< 0.01
					0.36 (0.21, 0.63)	< 0.01
Moderate/High MRI suspicion	4	365/554	0.61	0	1.46 (1.28, 1.67)	< 0.01
					1.43 (1.27, 1.63)	< 0.01
Clinically significant PCa	10	2481/2583	0.46	0	1.19 (1.10, 1.29)	< 0.01
					1.19 (1.10, 1.28)	< 0.01
Clinically insignificant PCa	10	2395/2494	0.08	43	0.68 (0.59, 0.79)	< 0.01
					0.66 (0.51, 0.86)	< 0.01

CI = confidence interval; RD = risk difference.

P_h_ values for heterogeneity from Q-test.

* MR/US fusion biopsy sample size/System biopsies sample size

a Fixed effect model was used when heterogeneity P_h_ >0.05.

b Random effect model was used when heterogeneity P_h_ <0.05.

### Comparison of MR/US fusion and systematic cores for the detection of prostate cancer

Seven trials were used to investigate this question. For core-by-core analysis, 1536 of 5777 MR/US biopsy cores (26.6%) were positive. In contrast, only 1866 of 18221 systematic biopsy cores (10.2%) were positive, resulting a RR of 2.75 (95% CI, 2.58–2.92; *p* < 0.01). However, there was heterogeneity among these Trials (I^2^ = 96%; x^2^ = 166.48; *p* < 0.01).

### Comparison of the two biopsy protocols in the detection of clinically significant prostate cancer

**Figure 6:**
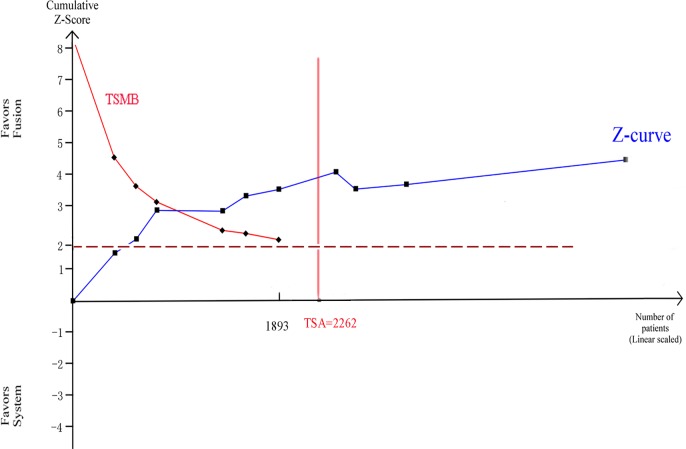
Trial sequential analysis of trials of two biopsy strategies in detecting clinically significant prostate cancer Number of participants reaches the information size and the cumulative Z-curve crosses the traditional significance boundaries and TSMB.

Ten trials were used to perform this analysis. Clinically significant prostate cancer was diagnosed in 892 of the 2481 men with MR/US fusion biopsy compared with 786 of the 2583 men with TRUS-guided systematic biopsy, resulting in a RR of 1.19 (95% CI, 1.10–1.29; *p* < 0.01; Table 3), and there was no heterogeneity among these trials (I^2^ = 0%; x^2^ = 8.77; *p* = 0.46). Clinically insignificant prostate cancer was diagnosed in 255 of 2395 men with MR/US fusion biopsy compared with 368 of 2494 men with TRUS-guided systematic biopsy, resulting in a RR of 0.68 (95% CI, 0.59–0.79; *p* < 0.01; Table 3), and there was high heterogeneity among these Trials (I^2^ = 72%; x^2^ = 63.78; *p* < 0.01). For trial sequential analysis, the number of participants did reach the information size and the cumulative Z-curve crossed both the traditional significance boundaries and TSMB (Figure 6).

## DISCUSSION

Systematic prostate biopsy using TRUS has been the gold standard technique for detection of prostate cancer. However, there has been a recent emphasis on discrepancies between the results of systematic prostate biopsies and radical prostatectomy specimens [15]. Systematic biopsy also tends to miss clinically significant prostate cancers, which might delay the treatment of a tumor with a high Gleason score. MR/US fusion prostate biopsy is reportedly a better choice that saves both time and money.

Our study showed that MR/US fusion biopsy detected more prostate cancers than systematic biopsy (46.9% *vs* 44.2%, *p* = 0.03). Our findings differ from the preceding systematic reviews [9,10], perhaps because we used larger sample sizes and updated data [11]. Trial sequential analysis was largely uninformative because of the low numbers, but suggested that more evidence is needed to confirm this outcome. We used quantified Q and I^2^ tests to assess the degree of heterogeneity across the eligible studies. We found that there was moderate overall heterogeneity among these trials (I^2^ = 28%; x^2^ = 20.92; *p* = 0.14), though Egger's test and funnel plots indicated very little publication bias.

In subgroup analysis, we found that with an mp-MRI lesion considered of moderate/high suspicion, MR/US fusion biopsy was better than systematic biopsy at achieving a correct diagnosis with fewer core samples (56.7% *vs* 39.5%, *p* < 0.01). The criterion used to delimit the lesions in mp-MRI included PI-RADS scores and NIH MP-MRI scores, among others (Table 1). The details of the PI-RADS scoring and NIH MP-MRI scoring are listed in Table 4. For our analysis, we defined a PI-RADS score of 2 or 3 as MRI low suspicion, a score of 4 as MRI moderate suspicion, and a score of 5 as MRI high suspicion. There are several limitations to this subanalysis. First, only four trials with 554 patients were included, resulting in a limited sample size. Second, there was heterogeneity in the low MRI suspicion group analysis due to inter-reader variability and limited sample size. Although the PI-RADS and NIH MP-MRI scoring systems reportedly improve inter-reader agreement, this remains to be confirmed. One study by Vourganti and colleagues [16] did not follow the recommendations, and mp-MRI sequences were reviewed by radiologists who identified and graded lesions on suspicion for cancer, which might result in more inter-reader variability.

When compared to radical prostatectomy histology, mp-MRI was reported to have a high diagnosis rate of clinically significant prostate cancer [17]. It was most sensitive at detecting tumors larger than 5 mm in diameter and/or with Gleason scores of 8 or greater [18]. Moore and colleagues [19] also reported that the efficiency (i.e., number of clinically significant prostate cancer/number of men biopsied) of the MRI targeted biopsy outperformed the standard approach (70% *vs*. 40%). According to our subgroup analysis, which was consistent with earlier studies, in men with mp-MRI of moderate/high MRI suspicion, MR/US fusion biopsy was effective. For trial sequential analysis, the cumulative Z-curve crossed the monitoring boundaries constructed from both information size calculations, thereby confirming that MR/US fusion biopsy is better than traditional systematic biopsy in detecting clinically significant prostate cancer.

MR/US fusion and systematic approaches can be compared on either a per-patient or a per-core basis. The latter allows some assessment of the potential efficiency of a new approach [19]. For core-by-core analysis, MR/US biopsy outperformed systematic biopsy, requiring fewer cores to make a successful diagnosis. However, there was significant heterogeneity among these trials and a limited sample size. Previous reports showed that the number of sampled cores does not correlate with complications [20-22]; however, a prostate biopsy with fewer cores is less uncomfortable for patients.

Targeted biopsy as an adjunct to the standard 12-core biopsy resulted in Gleason score upgrades compared with 12-core biopsy alone. Siddiqui and colleagues [23] found a 32% rate of Gleason score upgrading (81 of 255) in prostate cancer cases diagnosed on 12-core biopsy alone, and Vourganti and colleagues found a rate of 38.4% (28 of 73) in prostate cancer cases diagnosed on 12-core biopsy alone. Shakir and colleagues [24] also reported that above a PSA threshold of 5.2 ng/ml there were more upgrades to clinically significant prostate cancer by MR/US fusion biopsy compared with systematic biopsy. There is a significant correlation between Gleason score and the prognosis of cancer [25]. Because MR/US fusion biopsy tends to assign a more precise Gleason score than systematic biopsy, this could lead to better, faster treatment in men with prostate cancer.

Our study had several limitations. First, although our analysis revealed no publication bias, we only searched a limited number of databases and did not retrieve unpublished studies. Second, for a suspicious lesion detected with mp-MRI, the cancer detection rate is contingent on sampling density [19]. The precise density was hard to calculate for the included studies because the volume of the target of the fusion biopsy was never published. Third, there is no well-accepted standardized scoring system for suspicion in mp-MRI, and trials included in the subgroup analysis had different criteria. We divided the scores into low and moderate/high suspicion for our analysis, which might lead to bias. Fourth, bias existed in the studies because fusion biopsies and systematic biopsies were taken from the same patient. In the biopsy protocol, fusion sampling was undertaken first, which may lead to a systematic sample of a different volume of tissue. Even worse, if the prior needle tracks were avoided, the detection rate of systematic cores would inevitably be lower. Finally, trial sequential analysis cannot fix the error caused by the methodology of the trials. Because the TRUS-guided prostate biopsy was chosen as reference standard, which was not likely to correctly classify the target condition, the included studies did not have a low risk of bias.

In summary, we analyzed the current high-level clinical evidence to evaluate the MR/US fusion prostate biopsy. We found that, although more evidence is needed, MR/US fusion prostate biopsy alone detected more prostate cancers than systematic biopsy and was better than systematic biopsy in detecting clinically significant prostate cancers. For those men with moderate/high suspicion in mp-MRI, MR/US fusion biopsy showed a great advantage. We therefore recommend that mp-MRI should be performed in men suspected of having prostate cancer. For those men with moderate/high suspicion in mp-MRI, MR/US fusion biopsy is a better choice.

**Table 4:** The details of PI-RADS scoring and NIH MP-MRI scoring system

PI-RADS scoring system		NIH MP-MRI scoring system	
Score 1	Clinically significant disease is highly unlikely to be present	Low risk	positive on 1 or 2 of the 3 sequences (triplanar T2-weighted, axial
Score 2	Clinically significant cancer is unlikely to be present		diffusion weighted with ADC mapping and dynamic contrast enhanced)
Score 3	Clinically significant cancer is equivocal	Moderate/high risk	all 3 parameters were positive
Score 4	Clinically significant cancer is likely to be present	High risk	all 4 parameters are positive, including MR spectroscopy
Score 5	Clinically significant cancer is highly likely to be present.		

## MATERIALS AND METHODS

### Systematic search strategy

We conducted a systematic search of electronic databases, including the Cochrane Library, Embase, Web of Knowledge, and Medline (updated to May 1, 2015), to identify all relevant studies. The search terms used were listed as follows: ‘multiparametric magnetic resonance imaging OR mp-MRI OR TRUS’ AND ‘prostate biopsy OR target biopsy’. Abstracts were reviewed for relevance to the defined review question. If it was not clear from the abstract whether the paper contained relevant data, the full paper was assessed in the next step. The references cited in all full-text articles were also assessed for additional relevant articles. Three independent authors were included throughout the systematic search. The search was carried out independently for each database by two of them. If there was any disagreement the third author would arbitrate.

### Inclusion and exclusion criteria

Trials conducted to compare TRUS-guided prostate biopsy and MR/US fusion guided prostate biopsy were included, regardless of whether they were first biopsy or repeated ones. The following criteria were also met: (1) the protocol of mp-MRI and MR/US fusion guided biopsy was as follows: the populations reported in the trials were referred with a clinical suspicion of prostate cancer (due to a raised PSA or an abnormal DRE), then underwent a diagnostic mp-MRI of the prostate. Suspicious areas were defined as previously reported [26]. Lesions suspicious for cancer identified on MRI were displayed on the real-time TRUS image. Fusion of MRI and real-time ultrasound was performed as described previously [27]. (2) Articles used to compare the detective rate of clinically significant prostate cancer between two biopsy protocols were listed clearly in the article. (3) Exact statistics of fusion biopsy and system biopsy group were identified. If more than one publication from the same population were available, the study with the largest number of cases was included. Trials with insufficient or overlapping data were excluded.

### Data extraction

The data were extracted from the articles by including name of author, year of publication, country, participant details (number of patients and pre-biopsy parameters), biopsy details (different core number), definition for clinically significant Prostate cancer, score used in mp-MRI, and results (detection rate of Prostate cancer and different detection rate between two biopsy methods) [28]. Two investigators extracted the data independently.

### Quality assessment

The quality of each trial was evaluated using the Quality Assessment Tool for Diagnostic Accuracy Studies (QUADAS-2) [29], which includes four domains: selection; index test conduct; reference test conduct; participant flow and timing. According to QUADAS-2 guidelines, a study was appropriate to have an overall judgment of “low risk of bias”, if it was judged as “low” on all domains relating to bias or applicability. A study might be judged to be at overall risk of bias if only 1 or more domains were judged “high” or “unclear”. The assessment was carried out by two authors. If there was any disagreement a third author would re-evaluate the original study.

### Trial sequential analysis

For repeated updates of meta-analysis, a new Z-value was calculated for each update. In the trial sequential analysis, Z-values were plotted against number of patients, outcomes, or information. Next, the cumulative Z-curve is assessed according to its relation to the traditional significant boundaries (Z = ±1.96), the required information size, and the trial sequential monitoring boundaries (TSMB).[30, 31] TSMB was calculated due to Lan-DeMets version of the O'Brien–Fleming function [14].

In trial sequential analysis, the parameters were set as follows: risk of type I error α = 0.05; risk of type II error β = 0.20. Relative risk reduction was calculated by incidence in intervention group and incidence in control group based on low-bias. TSA v0.9 (Copenhagen Trial Unit, Copenhagen, Denmark) was used for trial sequential analysis.

### Statistical analysis

We used risk ratio (RR) and 95% confidence intervals (CIs) to compare MR/US fusion prostate biopsy and system prostate biopsy. The Mantel-Haenszel estimates were used and pooled under a fixed or random effect model when appropriate. Quantified Q test [32] and I^2^ test [33] are used to evaluate the degree of heterogeneity across the included studies. Heterogeneity was confirmed with a significance level of *p* < 0.05. Studies with an I^2^ < 25% were considered as no heterogeneity; I^2^ = 25-50% as moderate heterogeneity; I^2^ >50% as large heterogeneity. The aforementioned analyses were performed by RevMan v.5.2. Additionally, the Egger's test and the Begg's test were used to evaluate the publication bias by STATA v.11.0 (StataCorp, College Station, TX, USA).

## SUPPLEMENTARY MATERIAL


